# Novel therapeutics in myeloproliferative neoplasms

**DOI:** 10.1186/s13045-020-00995-y

**Published:** 2020-12-02

**Authors:** Sangeetha Venugopal, John Mascarenhas

**Affiliations:** 1 Department of Leukemia, MD Anderson Cancer Center, University of Texas, Houston, TX, 77030 USA; 2 Division of Hematology/Oncology, Tisch Cancer Institute, Icahn School of Medicine at Mount Sinai, One Gustave L Levy Place, Box 1079, New York, NY 10029 USA

**Keywords:** MF, ET, PV, JAK-STAT, CALR, Ruxolitinib, Fedratinib, Pacritinib, Imetelstat, CALR vaccine

## Abstract

Hyperactive signaling of the Janus-Associated Kinase/Signal Transducers and Activators of Transcription (JAK/STAT) pathway is central to the pathogenesis of Philadelphia-chromosome-negative myeloproliferative neoplasms (MPN), i.e., polycythemia vera (PV), essential thrombocythemia (ET), and primary myelofibrosis (PMF) which are characterized by inherent biological and clinical heterogeneity. Patients with MPNs suffer from substantial symptom burden and curtailed longevity due to thrombohemorrhagic complications or progression to myelofibrosis or acute myeloid leukemia. Therefore, the management strategies focus on thrombosis risk mitigation in PV/ET, alleviation of symptom burden and improvement in cytopenias and red blood cell transfusion requirements, and disease course alteration in PMF. The United States Food and Drug Administration’s (USFDA) approval of two JAK inhibitors (ruxolitinib, fedratinib) has transformed the therapeutic landscape of MPNs in assuaging the need for frequent therapeutic phlebotomy (PV) and reduction in spleen and symptom burden (PV and PMF). Despite improving biological understanding of these complex clonal hematopoietic stem/progenitor cell neoplasms, none of the currently available therapies appear to modify the proclivity of the disease per se, thereby remaining an urgent unmet clinical need and an ongoing area of intense clinical investigation. This review will highlight the evolving targeted therapeutic agents that are in early- and late-stage MPN clinical development.

## Introduction

Polycythemia vera (PV), essential thrombocythemia (ET), and primary myelofibrosis (PMF) are clonal myeloproliferative neoplasms (MPN) with distinct hematological and clinicopathologic features that can be viewed as a disease spectrum [1]. Approximately 90% of patients with MPNs harbor mutations involving the *JAK2*, *CALR,* or *MPL* genes (phenotypic drivers in MPN), resulting in hyperactivation of the Janus-Associated Kinase/Signal Transducers and Activators of Transcription (JAK/STAT) signaling pathway [2–4]. Additionally, they may harbor mutations in the epigenetic modifiers (*DNMT3A, TET2, ASXL1, IDH1/2, EZH2*), RNA splicing (*SRSF2, U2AF1*), tumor suppressor (*TP53*) genes that co-operate with each other, and the driver mutations to bestow a more advanced disease phenotype [5]. While patients with PMF suffer from debilitating constitutional symptoms, progressive splenomegaly and cytopenias, PV and ET patients experience microvascular symptoms (headaches, erythromelalgia, Raynaud syndrome) and grievous life-threatening thromboses (arterial and venous) [6, 7]. In general, patients with MPN are at an increased risk of developing thrombosis compared to the general population (PV > ET > PMF) [8] and may progress to acute myeloid leukemia (PMF > PV > ET) [9]. Therefore, prevention of thrombosis and disease progression form the two-pronged approach in the treatment strategy of MPNs. While the current prognostic models in PV and ET are predicated on the clinical and hematological parameters that predict the risk of recurrent thrombosis [10, 11], the integration of molecular and clinical data in PMF has allowed for more refined risk stratification and early evaluation for hematopoietic cell transplantation (HCT) [12], which remains the only curative treatment modality.

## Agents in clinical development in PV and ET

Age (< 60 vs. > 60 years) and the history of thrombosis form the basis of the risk-adapted approach informing the management decisions in PV and ET as thrombosis is the leading cause of preventable death in these MPNs [11, 13]. Patients younger than 60 years of age with no history of thrombosis are categorized as “low risk” and managed conservatively with therapeutic phlebotomy to maintain a hematocrit less than 45% in PV [14]. In both PV and ET, these low-risk patients are counseled to optimize cardiovascular risk factors (smoking, blood pressure, obesity) and prescribed low-dose aspirin for thrombosis prevention except in *JAK2* wild-type ET patients who are deemed as very low risk and maintained on observation only [15]. Cytoreductive therapy is reserved for those patients with “high-risk” features in both ET and PV and low-risk PV patients suffering from uncontrolled symptoms, symptomatic splenomegaly, and intolerance to therapeutic phlebotomy. While hydroxyurea (HU) is the initial drug of choice [16], [17] pegylated interferon-α (IFNα) is preferred in younger patients desiring offspring as HU is a potential teratogen [18, 19] and ruxolitinib, a JAK1/2 inhibitor for those PV patients who are intolerant or resistant to HU [20, 21].

Although low-risk PV patients are managed with therapeutic phlebotomy and aspirin, they still experience higher than normal rates of thrombosis compared to the general population [8] as they may not have a well-controlled hematocrit (< 45%) between visits that may predispose them to poor outcomes secondary to hyperviscosity. Additionally, therapeutic phlebotomy leads to iron deficiency-related symptoms that may exacerbate/mimic PV-related symptoms. In this regard, hepcidin mimetics are being evaluated in PV as an alternative to therapeutic phlebotomy as hepcidin regulates iron metabolism, limits intestinal iron absorption, and restricts erythropoiesis. Preclinical studies of minihepcidin in murine models of PV have shown that prolonged administration curbs the availability of iron to erythroid precursors, thereby impeding erythropoiesis, resulting in the normalization of hematocrit [22]. PTG300, a self-injectable hepcidin mimetic administered weekly, is currently being evaluated as a “medical phlebotomy agent” in a phase II study in PV patients requiring frequent therapeutic phlebotomy (NCT04057040).

Given the inherent risk of thrombosis in PV regardless of the current risk stratification, the move to initiate cytoreductive therapy to mitigate the risk of thrombosis in low-risk PV patients is gaining momentum. IFNα may have disease-modifying activity in MPNs as evidenced by preclinical studies; several small phase 2 studies have shown that IFNα treatment can induce molecular and cytogenetic responses in treated MPN patients, although the results vary according to the series. While some investigators have reported that patients harboring TET2 co-mutations do not respond as well to IFNα as those harboring wildtype, others have reported that patients with low *JAK2* V617F variant allele frequency (VAF) are more likely to achieve complete hematological response with IFNα treatment than those with higher VAF at baseline [23–26]. Nevertheless, IFNα appears to induce hematological, molecular, and cytogenetic responses [27] and the clinical benefit of IFNα appears to be optimal when employed earlier in the disease course. In this context, the LOW PV trial is evaluating Ropeginterferon alfa-2b (Ropeg) compared to therapeutic phlebotomy in a phase II randomized clinical trial in low-risk PV patients. Ropeg is a monopegylated interferon that overcomes the shortcomings of IFNα (administered weekly), allowing for less frequent dosing (administered every two weeks) and improved patient tolerability ensuring long-term patient compliance [28]. Maintaining hematocrit ≤ 45% for 12 months in the absence of progressive disease is the primary composite endpoint of the LOW PV trial. The recently presented preplanned interim analysis shows that 84% of patients on the ropeg arm achieved the primary composite endpoint (60% in the phlebotomy arm; OR = 3.5, 95% CI 1.3–10.4, *p* = 0.008) with a lower number of required therapeutic phlebotomy after one year of treatment (43% vs. 57%; *p* = 0.024). Ropeg was well tolerated with no significant difference in adverse events (AE) between both treatment arms. This trial has stopped enrollment in view of the resounding efficacy, and follow-up will continue for 2 years per protocol [29]. The final results of this trial may have practice-changing implications in patients with low-risk PV.

Previously, the Myeloproliferative Disorders Research Consortium (MPD-RC) 112 study and MPD-RC 111 study have highlighted the activity of IFNα in treatment-naïve and HU-resistant/refractory ET/PV patients, respectively [26, 30]. Most recently, phase III randomized controlled trials, PROUD-PV and its extension study CONTINUATION-PV, evaluated ropeg against HU in patients with PV. PROUD-PV was powered to establish the non-inferiority of ropeg against HU with a composite primary endpoint of complete hematological response (CHR) and resolution of splenomegaly at 12 months; CHR and symptomatic improvement were the coprimary endpoints in the CONTINUATION-PV study. At a median follow-up of 182 weeks in the ropeg arm, 21% (28% in HU arm at a median follow-up of 164 weeks) and 53% of patients (38% in HU arm, *p* = 0·044) met the primary endpoints in PROUD-PV and CONTINUATION-PV study, respectively. CHR without the spleen criterion in the ropeg arm was met in 43% (46% in HU, *p* = 0·63 at 12 months) and 71% (51% in HU, *p* = 0·012 at 36 months) in the PROUD-PV and CONTINUATION-PV, respectively. Liver enzyme abnormalities were the most frequently reported grade 3/4 adverse events in the ropeg arm and expected myelosuppression in the HU arm with comparable rates between the groups. Neuropsychiatric manifestations in the ropeg arm were rare [31]. Given these encouraging results, Ropeg is currently approved in Europe as a first-line agent for the treatment of PV in the absence of symptomatic splenomegaly and is under review for FDA approval in the USA.

Givinostat, a histone-deacetylase (HDAC) inhibitor has demonstrated preclinical activity in selective targeting of the *JAK2* V617F clone by inhibiting the downstream signaling [32]. Subsequently, several studies have shown that givinostat is clinically active either as monotherapy or in combination with HU [33, 34]. Most recently, givinostat was evaluated in a dose-finding/proof of concept study in patients with PV. Givinostat exhibited on-target activity, and 100 mg twice daily was deemed the recommended phase 2 dose (RP2D). In part B, proof of concept phase, the ORR rate was 80.6% at the end of three cycles and 50% of patients reported symptomatic improvement (pruritus, headache) with givinostat treatment. Almost all patients experienced grade 1/2 treatment-related adverse event (TEAE) [diarrhea—51%; thrombocytopenia—45%; increased serum creatinine—37%]. Based on these results, a registration trial of givinostat in PV patients is underway [35].

The P53-MDM2 axis is a novel therapeutic target in MPNs. MDM2 negatively regulates p53, promotes its degradation as well as inhibits *p53* transcription. Preclinical studies have shown that MDM2 is upregulated in *JAK2* V617F-positive MPN hematopoietic progenitor cells, resulting in low p53 RNA levels that has led to the evaluation of MDM2 inhibitors in MPNs [36]. A recently published proof of concept study of Idasanutlin, an oral MDM2 inhibitor, in the second-line setting in patients with high-risk PV/ET demonstrated an overall response rate (ORR) of 58% (7/12) and a durable response (16.8 months) with monotherapy. Idasanutlin was well tolerated with no dose-limiting toxicities; low-grade gastrointestinal toxicity (diarrhea/nausea in 80%) was common but manageable with a scheduled antiemetic regimen. Collectively, idasanutlin demonstrated safety and on-target clinical activity in JAK inhibitor-naïve, HU/IFN-resistant, or intolerant PV/ET patients. A global phase II trial in HU refractory PV is underway [37]. (NCT03287245) Following suit, KRT232, a potent small molecule oral MDM2 inhibitor, is being evaluated as a second-line agent in phlebotomy-dependent PV patients (NCT03669965).

Lysine-specific demethylase 1 (LSD1) is an epigenetic enzyme that maintains steady-state hematopoiesis and LSD1 inhibition-abrogated erythropoiesis, granulopoiesis, and thrombopoiesis in a reversible fashion. Additionally, LSD1 is found to be overexpressed in MPNs [38]. IMG7289 (bomedemstat), an LSD1 inhibitor, reduced splenomegaly, normalized blood counts, and prolonged survival in the Jak2 V617F murine model [39], which has led to the clinical evaluation of bomedemstat as a second-line agent in PV and ET (NCT04254978) (NCT04262141).

Furthermore, the recent understanding of the mechanistic basis of *CALR* mutated MPN has revealed several potential novel therapeutic targets, especially in harnessing host immunity. *CALR* mutations generate a novel positively charged C terminus in the CALR protein, which could be exploited as a potential shared neoantigen, as the physical interaction between CALR and MPL is essential for *CALR*-induced myeloproliferation [40, 41]. Additionally, studies have shown that *CALR* is immunogenic and immune escape occurs in patients with *CALR*-mutated MPN [42]. In this regard, *CALR*-specific CD4 + T-cell clone, which demonstrated specific cytotoxicity against autologous *CALR*-mutant cells, has been generated [43], and these results have formed the basis of a phase 1 CALR exon 9 peptide vaccine in *CALR*-mutated MPNs. (NCT03566446) Most recently, Bozkus et al*.* demonstrated that a subset of patients with *CALR*-mutated MPN exhibits specific T-cell responses against the *CALR* C-terminus that is completely abrogated by the expression of PD-1 or CTLA4. Ex vivo treatment with an anti–PD-1 antibody restored mutant CALR-specific T-cell responses in the peripheral blood mononuclear cells of *CALR*-mutated MPN patients [44]. Clinical evaluation of a vaccine-based approach in combination with a PD-1 inhibitor is underway**.**

## Agents in clinical development in MF (Fig. 1)

**Fig. 1:**
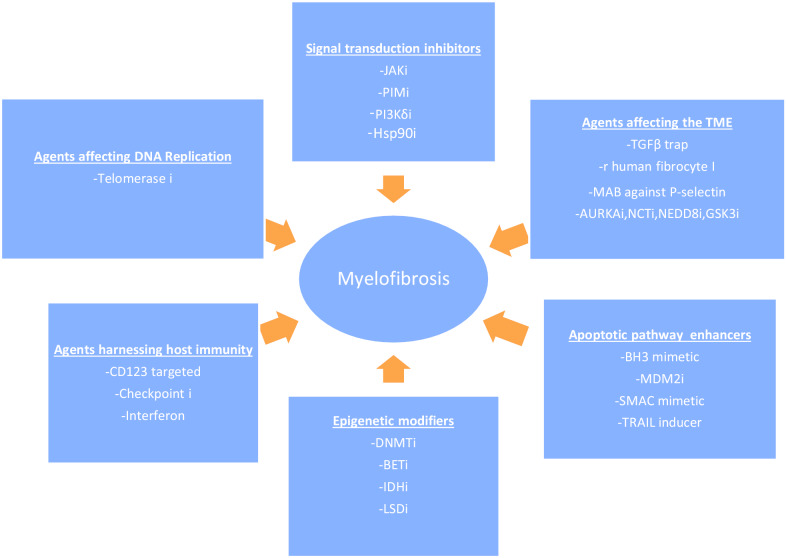
Agents in clinical development in MF. i—inhibitor; JAK—Janus-associated Kinase; PIM—Proviral Integration Site for Moloney Murine Leukemia Virus; PI3K—Phosphatidylinositol 3-Kinase; TGF—tumor growth factor; Hsp—heat-shock protein; AURKA—Aurora Kinase A;GSK—glycogen synthase kinase; NCT—nuclear-cytoplasmic transport; MAB—monoclonal antibody; NEDD—neddylation; BH—B-cell lymphoma homology; MDM—murine double minute; SMAC—second mitochondria-derived activator of caspases; TRAIL—tumor necrosis factor-related apoptosis inducing ligand; DNMT—DNA methyl transferase; IDH—isocitrate dehydrogenase; BET—bromodomain and extra-terminal motif; LSD1—lysine-specific demethylase 1

**Fig. 2:**
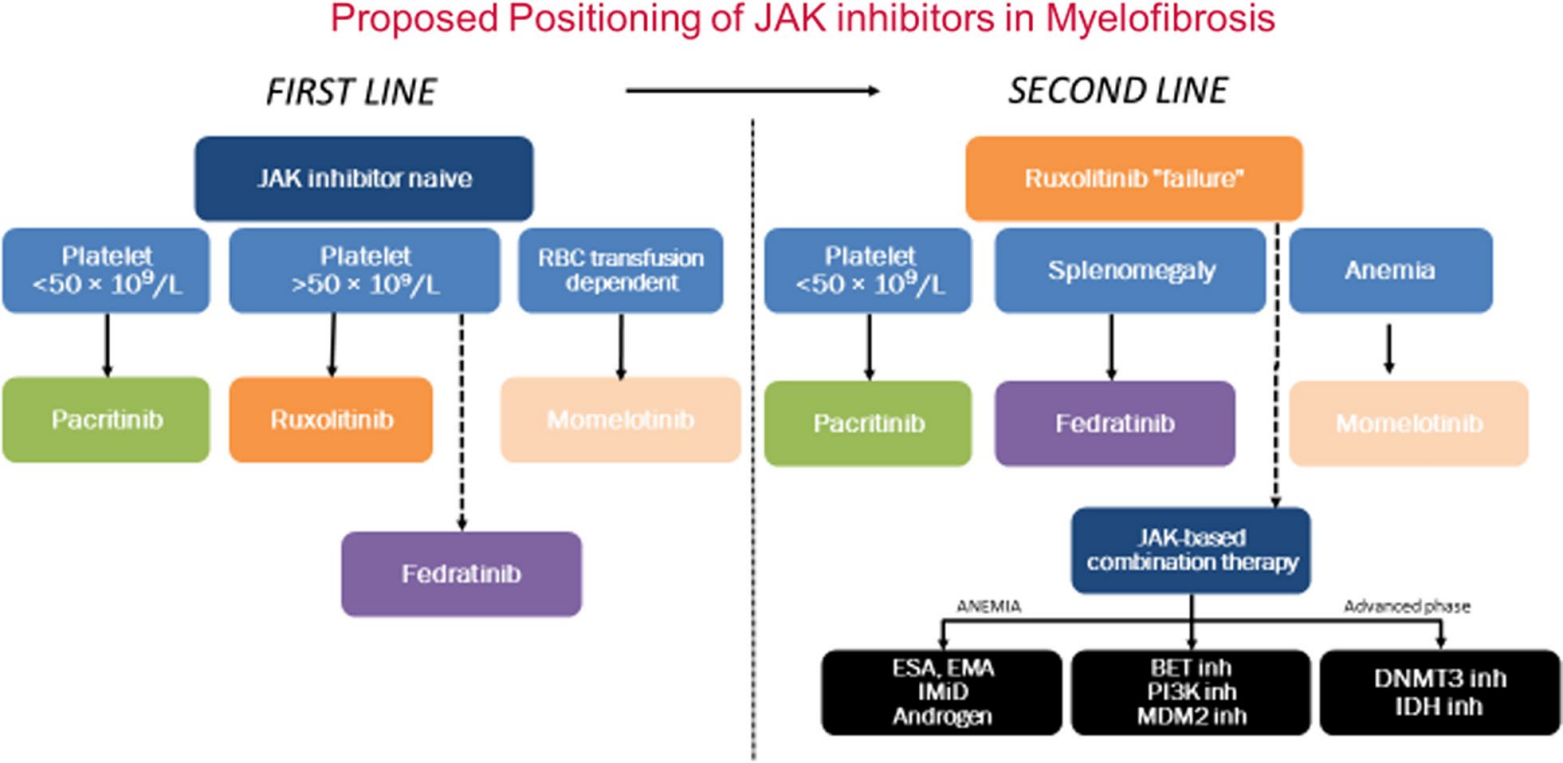
Positioning of JAK inhibitors in the treatment schema of myelofibrosis. ESA—erythropoiesis-stimulating agent; EMA—erythroid maturation agent; IMiD—immunomodulatory imide drugs; BET—Bromodomain and Extraterminal domain Protein; PI3K—Phosphatidylinositol 3-Kinase; MDM—murine double minute; IDH—isocitrate dehydrogenase

Ruxolitinib, a JAK1/JAK2 inhibitor (2011) [45] and fedratinib, a JAK2/FLT3 inhibitor (2019) [46] are approved in the USA for MF patients with splenomegaly and/or constitutional symptoms regardless of the presence of mutated *JAK2*. Although long-term follow-up studies have validated the sustained benefit of ruxolitinib in MF patients in terms of improvement in splenomegaly, symptom burden, and quality of life with an increase in overall survival (OS), a subset of patients are intolerant or refractory to JAK inhibitor therapy. While the median OS in ruxolitinib-treated patients is 60 months, the median OS post ruxolitinib discontinuation drops significantly (14 months) [47, 48]. Furthermore, clonal evolution or the finding of platelets < 100 × 10^9^/L at the time of ruxolitinib discontinuation was found to be associated with particularly poor prognosis in patients with MF. Additionally, Kuykendall et al*.* evaluated the clinical outcomes and salvage treatment options in patients who received and discontinued ruxolitinib. In 64 evaluable patients, new cytopenias (anemia—33%; thrombocytopenia—11%) were the most common reasons for an impediment to ruxolitinib continuation after a median treatment time of 3.8 months. Of note, 26% of patients responded to salvage treatment options leading to better outcomes than those who did not receive additional therapy, suggesting that responses were salvageable in some patients even after ruxolitinib discontinuation. However, these responses were rare, representing an area of unmet clinical need in ruxolitinib pretreated patients with MF [49]. Therefore, there is a constant drive to improve upon the existing treatment options in patients with MF. Currently, many novel therapeutic agents are in clinical development in the front-line setting (monotherapy), “Add on” with ruxolitinib as a complementary therapeutic strategy, second-line setting, or treatment directed at mitigation of cytopenias (Fig. 2).

### Front-line setting

Pacritinib is a multikinase inhibitor of JAK2, FLT3, IRAK1, and CSF1R, with less myelosuppressive effect noted in the early-phase trials and further evidenced by the anemia response [25% achieved transfusion independence (TI)] and platelet improvement (35% increase in mean platelet count noted in those with a baseline platelet count lower than 50 × 10^9^/L) in the PERSIST-1 randomized controlled trial in JAK inhibitor naïve patients with MF [50]. PERSIST-2, a randomized controlled trial, evaluated pacritinib in MF patients with either disease or therapy-related (ruxolitinib) thrombocytopenia (platelets < 100 × 10^9^/L), and they were randomized to two doses of pacritinib (200 mg BID or 400 mg once daily) or BAT (best available therapy), which could include ruxolitinib as well. Eighteen percent of patients enrolled on the pacritinib arms achieved ≥ 35% spleen volume reduction (SVR35%) compared with 3% on the BAT arm (*p* = 0.001), and these improvements were more noticeable in the pacritinib 200 mg BID arm [≥ 35% SVR: 22% vs. 3%; *p* = 0.001; ≥ 50% reduction in myelofibrosis-related total symptom score (TSS50): 32% vs. 14%; *p* = 0.01]. Grade 3 or more thrombocytopenia, cardiac AEs, and therapy discontinuation were less frequent in the twice-daily arm [51]. Pacritinib development was interrupted due to the full clinical hold placed by the FDA in February 2016 due to safety concerns (increased hemorrhagic risk and mortality), which prompted an independent review that deemed mortality rates were not different between the study arms. Recently presented phase II PAC203 (NCT03165734) dose-finding (100 mg daily, 100 mg twice daily, and 200 mg twice daily) study evaluated pacritinib with preplanned built-in safety protocols for mitigating cardiac and hemorrhagic risk (concomitant anticoagulant/antiplatelet and QT-prolonging agents were contraindicated). Pacritinib was well tolerated, and 17% of patients with severe thrombocytopenia (< 50 × 10^9^/L) attained spleen responses in the 200 mg BID cohort [52]. Given that thrombocytopenia (especially platelet count < 50 × 10^9^/L) is a poor prognostic factor in patients with MF and ruxolitinib is only approved for those with a minimum platelet count of 50 × 10^9^/L, pacritinib can potentially bridge this chasm and offer a viable therapeutic option for this challenging population subset. The PACIFICA phase III registration trial will evaluate the safety and efficacy of 200 mg BID of pacritinib compared to the physician's choice (low-dose ruxolitinib, corticosteroids, hydroxyurea, or danazol), in patients with MF and severe thrombocytopenia (< 50 × 10^9^/L) and less than 12 weeks of prior JAK inhibitor therapy [53] (NCT03165734).

### Ruxolitinib “Add-on” strategies

Itacitinib is a selective JAK1 inhibitor being evaluated in MF under the premise that selective JAK1 inhibition will abrogate proinflammatory signaling without affecting the JAK2-mediated hematopoiesis. A phase II open-label study evaluated the safety and efficacy of three dose levels [100 (*n* = 10) or 200 mg BID [45], 600 mg QD [32]] of itacitinib in MF patients with TSS50 at week 12 as the primary endpoint. In total, 35.7% and 35.5% achieved the primary endpoint in the 200 mg BID and 600 mg QD as compared to 20% in the 100 mg BID cohort. Modest SVR was observed in the higher dose cohorts. Notably, 53.8% experienced a ≥ 50% reduction in the number of red blood cell units transfused, and fatigue was the most common TEAE [54]. Itacitinib is currently being evaluated in two cohorts with one cohort in combination with ruxolitinib and the other in JAK inhibitor failure/intolerance in patients with MF (NCT03144687).

Masarova et al*.* investigated the sequential combination of ruxolitinib with azacitidine, a hypomethylating agent, preceded by an initial run-in phase with ruxolitinib monotherapy. In total, 72% (33/46) of patients achieved an objective response per International Working Group for Myelofibrosis Research and Treatment (IWG-MRT) criteria with a median time to response of 1.8 months, and most responses occurred on ruxolitinib monotherapy. In total, 57% of patients experienced ≥ grade 1 improvement in bone marrow fibrosis (BMF). Of note, 20% (3/15) of patients with cytogenetic abnormalities at diagnosis achieved a complete cytogenetic response at the end of 12 months of combination therapy. The combination was relatively well tolerated with only transient grade 3/4 myelosuppression that did not warrant treatment interruption [55] (NCT01787487).

CPI-0610 is a novel Bromodomain and Extraterminal domain Protein (BET) inhibitor that is currently undergoing clinical evaluation with two treatment arms investigating the “add-on” approach with ruxolitinib in MF patients in the JAK inhibitor naïve [Arm(A)3] and experienced (A2) with suboptimal response settings and the other evaluating CPI-0610 monotherapy in patients after ruxolitinib discontinuation (A1). In the first-line setting (A3), 73% achieved SVR35% at 12 weeks; 59% achieved TSS50, and 46% experienced at least one-grade improvement in BMF. In the JAK inhibitor “experienced” cohort, patients were stratified by red blood cell transfusion status. While 34% of evaluable transfusion-dependent (TD) patients converted to TI in A2 (NCT02158858), 21% of TD patients converted to TI in A1 and SVR35% was comparable in A1 (24%) and A2 (22%). CPI-0610 was tolerable with minimal grade 3/4 myelosuppression, and thrombocytopenia, low-grade nausea, and vomiting were the most commonly observed TEAE [56, 57]. Given these encouraging results (spleen and symptom response) and the potential disease-modifying activity (improvement in anemia and bone marrow fibrosis), a phase III, double-blind, randomized study comparing combination CPI-0610 and ruxolitinib to ruxolitinib monotherapy will start in the fourth quarter of 2020 (MANIFEST-2).

Phosphatidylinositol 3-Kinase (PI3K) inhibitors are being evaluated in combination with ruxolitinib to improve upon the suboptimal response to ruxolitinib in MF patients. In a phase II study of combination umbralisib and ruxolitinib therapy in MF, 9% (2/23) of treated patients achieved a complete response (CR), and 48% (11/23) experienced clinical improvement. One patient experienced colitis, but other class-specific side effects (hepatotoxicity, pneumonitis) were not observed [58]. The recently presented interim study results of parsaclisib, a potent and highly selective next-generation PI3Kδ inhibitor in combination with ruxolitinib. Parsaclisib was evaluated in two dosing schedules (QD for eight weeks followed by weekly; daily). Recently presented data showed that the intensive daily dosing schedule was found to be more efficacious than the weekly schedule (median percent change in spleen volume: − 13% vs. − 2.3%; TSS: − 51.4% vs. − 14.0%, respectively, at week 12]. Parsaclisib was well tolerated with no TEAE inherent to PI3K inhibitors (pneumonitis, colitis, diarrhea). Daily parsaclisib “add-on” to ruxolitinib will be evaluated further in a planned phase 3 trial (NCT02718300) [59].

Navitoclax, a non-selective Bcl2 inhibitor, is being evaluated in combination with ruxolitinib to improve response in patients with MF. These patients were heavily pretreated (> 3 lines of prior therapy), and 50% of treated patients harbored high molecular risk mutations (*n* = 34). Thirty percent of evaluable patients achieved SVR35%, 35% achieved TSS50, and ≥ 1-grade BMF reduction was seen in 25% of patients suggesting disease-modifying activity. On target thrombocytopenia was the most common TEAE, but there were no grade ≥ 3 bleeding events or treatment-related deaths. The combination was well-tolerated, and this combination will be evaluated in randomized phase 3 trials in both treatment-naïve and JAK inhibitor-treated patients [60] (NCT03222609).

A phase I/II RUXOPEG adaptive design trial is evaluating the combination of ruxolitinib and pegylated interferon alfa-2a in treatment-naïve DIPSS intermediate- or high-risk MF patients on the basis that this combination may permit administration of lower doses of interferon and improve tolerability. Phase I will test different combinations of three dose levels of each drug, and phase II will randomize the two best dose combinations from the phase I. The primary endpoint is composed of safety and efficacy objectives as denoted by the dose-limiting toxicity (DLT) within 45 days and SVR50% in 24 weeks, respectively. Thus far, fifteen patients have been enrolled in phase I; no DLT has been observed in the highest dose tested (ruxolitinib 15 mg BID + IFNa 135 mcg/week), and an early signal for efficacy has been reported (three-partial responses, seven-hematological improvement). The trial is ongoing (NCT02742324) [61].

### Second line: JAK inhibitor relapsed/ refractory/intolerant setting

The 5-year follow-up of the COMFORT-1 trial reported a median duration of approximately 3.2 years of spleen response, suggesting that the disease response to JAK inhibitors is not everlasting [62]. Although progressive disease per IWG-MRT includes only new/progressive splenomegaly and increasing blast counts either in the blood or marrow, clinically patients may exhibit evidence of disease progression through worsening cytopenias or loss of symptom response [63]. Currently, the widely accepted definition of ruxolitinib failure is centered around spleen size and presence of cytopenias (Table 1) [52]. Furthermore, a retrospective claims database study reported that the median treatment progression-free survival after ruxolitinib discontinuation is six months, 95% (CI: 4.4, 8.3 months), which, coupled with poor outcomes post discontinuation, reiterates the urgent need to explore novel therapeutic options in MF patients experiencing ruxolitinib failure (relapsed/refractory or intolerant to ruxolitinib treatment) [64].

**Table 1:** Criteria for ruxolitinib failure in patients with MF—adapted from [52, 65]

Criteria	Ruxolitinib duration	Cytopenias	Spleen size
Relapsed	≥ 3 months	–	Regrowth < 10% SVR or < 30% decrease in spleen size by palpation from baseline following an initial response
Refractory	≥ 3 months	–	< 10% SVR or < 30% decrease in spleen size by palpation from baseline
Intolerant	≥ 28 days	New grade ≥ 3 thrombocytopenia, anemia, hematoma/hemorrhage or RBC transfusion requirement ≥ 2 units/month for 2 months	–

*SVR* spleen volume reduction, *RBC* red blood cell

Momelotinib is a JAK1/2 inhibitor as well as a type 1 activin receptor (ACVR1) inhibitor being evaluated in MF patients with anemia on the premise that ACVR1 inhibition regulates hepcidin levels to restore iron homeostasis and improve anemia [66]. SIMPLIFY-1 study compared momelotinib with ruxolitinib in treatment-naïve MF patients. Although the trial met the non-inferiority primary endpoint for ≥ 35% SVR at 24 weeks (26.5% for momelotinib versus 29% for ruxolitinib, *p* = 0.011), it failed to meet the TSS50 endpoint. Notably, the momelotinib treatment arm enjoyed a higher rate of TI at week 24 than the ruxolitinib arm (66.5% vs. 49.3%, nominal *p* < 0.001) [67]. SIMPLIFY-2 compared momelotinib to BAT (including ruxolitinib) in MF patients intolerant to ruxolitinib. The study failed to meet its primary endpoint SVR35%, but the TSS50 endpoint was met. Akin to SIMPLIFY-1, more momelotinib-treated patients achieved TI (43% vs. 21% nominal *p* = 0.0012) [68]. However, in both trials, the hierarchal study design precluded the investigators from claiming the statistically significant anemia-related endpoints. Most recently, an open-label phase II study evaluated momelotinib in RBC TD patients with MF, and 34% achieved TI at week 24 [69]. The MOMENTUM trial will compare momelotinib to danazol in symptomatic and anemic patients with MF in the second-line setting (NCT04173494).

PRM-151 is a recombinant form of pentraxin-2, an endogenous serum amyloid protein that downregulates activated fibrogenic monocyte-macrophages activity in several organ models of fibrosis, including the bone marrow [70]. The first stage of phase II, open-label, extension study showed that PRM-151 was well tolerated as a monthly infusion either alone or in combination with ruxolitinib, and no unexpected AEs were observed in patients with MF. TSS50 was similar between both arms, and 44% of treated patients experienced at least a 1-grade reduction from grade 3 BMF at baseline (NCT01981850) [71]. In stage two, randomized, double-blind evaluation of three dose levels of PRM-151 infusional monotherapy in MF patients intolerant/refractory to JAK inhibitors, the primary endpoint was to determine the effective dose inducing at least a 1-grade reduction in BMF. All tested dose levels demonstrated greater than 1-grade BMF reduction, and the effect was similar across the tested doses [0.3 mg/kg: 30%; 3 mg/kg: 28%, and 10 mg/kg: 25%]. SVR35% was observed in only one patient. PRM-151 was well tolerated, and non-hematological AEs included fatigue, cough, and weight loss. Despite these encouraging findings, the further development of this drug in MF is uncertain as it evinced mostly BMF reduction. PRM-151 is currently undergoing registration trials in idiopathic pulmonary fibrosis [72].

Bomedemstat inhibits LSD1, an epigenetic target of interest in MPNs. LSD1 is essential for normal megakaryocyte function, and thrombocytopenia would be an expected dose-limiting side effect of LSD1 inhibition. In a phase II trial of bomedemstat in the second-line setting (*n* = 31), 12.5% of treated patients achieved SVR35%, 44.4% experienced TSS50, and ≥ 1-grade BMF reduction was noted in 15% of treated patients. Given the expectant thrombocytopenia, the dose up-titration of bomedemstat was individualized to achieve a target platelet count of 50 × 10^9^/L. No new safety signals or DLTs were observed. Further evaluation is underway (NCT03136185) [73].

Harnessing the targets in the apoptotic machinery has long been an object of clinical interest in MF. KRT-232, a potent oral MDM2 inhibitor, is currently under clinical investigation in the second-line setting in patients with advanced MF. This study excludes patients who are intolerant to ruxolitinib and does not require a ruxolitinib washout period [74]. Patients were randomly assigned to either of the three-dose arms (120 (A1) or 240 mg (A3) daily for seven days in a 21-day cycle or 240 mg daily (A3) for seven days in a 28-day cycle). As 16% of patients achieved SVR35% in A3, 240 mg daily for seven days in a 28-day cycle is deemed to be the RP2D for further evaluation. In total, 51% of treated patients experienced grade 3 TEAE with gastrointestinal symptoms being the most common AEs [diarrhea (62%) and nausea (38%)] [75] (NCT03662126). KRT-232 is now being evaluated as combination therapy with ruxolitinib in a phase 1/2 trial enrolling patients with suboptimal response to single agent ruxolitinib (NCT04485260).

LCL-161 is an oral second mitochondrial activator of caspases (SMAC) mimetic that inhibits apoptosis and is administered on a weekly basis. The phase 2 study included all comers with advanced MF in the second-line setting with no restrictions for platelet count or previous HCT. Among 47 evaluable patients, 32% ORR was observed with most response improvement in symptom burden and anemia; only one patient had a spleen response. Fatigue was the most common cause for dose reduction, and low-grade nausea/vomiting was observed in 60% of the patients [76] (NCT02098161).

Alisertib is an aurora kinase A (AURKA) inhibitor that promotes megakaryocyte differentiation in MF and may mitigate bone marrow fibrosis. Alisertib was evaluated in patients with advanced MF in the second-line setting with a minimum platelet count ≥ 50 × 10^9^/L and absolute neutrophil count ≥ 1 × 10^9^/L. Alisertib was well tolerated, and spleen and symptom improvement were observed in 29% and 32% of patients, respectively. Most importantly, alisertib normalized the atypical morphology of megakaryocytes (restored the multilobed nuclei and abrogated clustering), and among the seven patients with available sequential marrow samples, five patients experienced > 1-grade BMF, which correlated with the clinical responses (NCT02530619). The future development pathway for Alisertib is unclear [77].

Tagraxofusp is a CD123-targeted agent and currently approved in the treatment of blastic plasmacytoid dendritic cell neoplasm [78]. The shared phylogeny of plasmacytoid dendritic cells and monocytes, coupled with poor outcomes in MF patients with peripheral blood monocytosis, prompted the evaluation of tagraxofusp in the second-line setting. The study included all comers with no limitation in minimal platelet count at enrollment, and 26% of patients had documented monocytosis at baseline. Tagraxofusp was administered intravenously for three consecutive days in a 28-day cycle. Among 20 evaluable patients, 35% experienced objective clinical improvement, and 53% with baseline splenomegaly had some degree of reduction in spleen size as their best response. Tagraxofusp was reasonably well tolerated, with one patient experiencing grade 3 capillary leak syndrome [79] (NCT02268253).

Imetelstat is a competitive inhibitor of the telomerase enzyme complex comprising the RNA template with reverse transcriptase activity (hTERT). In a proof of concept study of 33 patients with advanced MF, imetelstat evinced an ORR of 21% limited to those with *JAK2, SF3B1*, or *U2AF1* mutations. The study did not show on-target activity (telomerase length) [80]. The subsequent phase 2, global IMBARK trial evaluated two dose levels of imetelstat (4.7 mg/kg and 9.4 mg/kg) administered intravenously every three weeks in 107 patients with advanced MF in the second-line setting (NCT02426086). Although SVR35% (10%) and TSS50 (32%) were only modest in the higher dose arm, the median survival was 29.9 months as compared with 19 months in the low-dose arm and the reported median survival of 13–14 months following ruxolitinib discontinuation [81]. Furthermore, imetelstat exhibited on-target activity and brought about greater than 50% reduction of hTERT expression levels, which correlated with clinical responses and longer OS in the 9.4 mg/kg arm [82]. Most importantly, the survival advantage of imetelstat was validated in a real-world cohort using a closely matched propensity score analysis [30.69 mo (95% CI 25.2, not estimable) vs. 12.04 mo (95% CI 9.5, 16.6) (BAT)] [83]. Given these encouraging results, the phase III registration trial of imetelstat is soon underway with OS as the primary endpoint, a novel endpoint that has never been explored in the drug development landscape of MF.

### Drugs targeting cytopenias in MF

Anemia independently predicts shortened survival in MF, and TD-anemia categorizes an MF patient in the higher-risk category regardless of the presence or absence of other adverse risk factors [84]. Furthermore, anemia is the most common reason for ruxolitinib discontinuation [49]. Several drugs are in clinical development for mitigating anemia in MF so as to safely continue MF-directed therapy. MPNSG-0212, a German study, evaluated pomalidomide in combination with ruxolitinib [two dose levels of pomalidomide: fixed low dose (A1) and dose escalation up to 2 mg (A2)] in MF patients with anemia ± RBC-TD. The A1 cohort exhibited an ORR of 18%, and there was a trend to sustained hemoglobin improvement with longer durations of treatment. TEAE was comparable between both arms with pneumonia and sepsis being the most grade ≥ 3 AEs [85].

Sotatercept and luspatercept are erythroid maturation agents (EMA) that act as activin receptor ligand traps of IIA and IIB, respectively [86, 87]. They are administered subcutaneously every three weeks, and luspatercept is currently approved for the treatment of anemia in low-risk myelodysplastic syndrome with ringed sideroblasts [88]. Sustained hemoglobin increase ≥ 1.5 g/dL for ≥ 12 consecutive weeks in TI patients or achieving RBC-TI in TD patients is the primary endpoints in the clinical trial evaluation of these EMAs in MF. Sotatercept monotherapy demonstrated an ORR of 35%, of which three patients achieved RBC-TI [89] (NCT01712308). In the recently presented study of luspatercept monotherapy and combination therapy with ruxolitinib in anemic patients with MF, 10% of treated patients each in the luspatercept monotherapy arm and 21% and 32% in the combination therapy arm achieved the primary endpoint in TI and TD patients, respectively (NCT03194542) [90]. Hypertension and bone pain were the most common, class-specific TEAE shared by both drugs. Further evaluation is ongoing, and a phase 3 trial is being planned.

Disease-related thrombocytopenia is an adverse prognostic factor in MF, which often precludes these patients from treatment with a JAK inhibitor or leads to dose attenuation resulting in suboptimal responses. Thalidomide in combination with prednisone has evoked modest improvement in platelet counts in patients with MF [91]. Most recently, a study of low-dose thalidomide in combination with ruxolitinib in patients with MF in the second-line setting (relapsed/refractory) showed an ORR of 60%, and platelet response was observed in 75% of patients with baseline thrombocytopenia. The combination was well tolerated with one patient each experiencing a thromboembolic event and grade 3 neutropenia. This combination may allow for optimal dosing of ruxolitinib in MF patients with baseline thrombocytopenia [92].

Several other agents exploiting the interconnected pathological pathways in MF are in various stages of early-phase clinical development (Table 2).

**Table 2:** Agents currently in early phase of clinical development in myelofibrosis

Drug	Mechanism of action	Setting	End points	Status	NCT id	References
PIM447 and LEE011	pan-PIM inhibitor, and CDK4/6 inhibitor	Add on to ruxolitinib phase 1b	Incidence of DLTs	A	NCT02370706	[93]
Enasidenib	IDH2 inhibitor	Add on phase 2	2^0^—Proportion of patients with any response^*^	NYR	NCT04281498	[94]
APG-1252	parenteral BH3 mimetic	Add on phase 1b/2	DLT at each dose level; SVR35% or TSS50	NYR	NCT04354727	–
PU-H71	Epichaperome-specific Hsp90 inhibitor	Add on phase 1b	–	Terminated as of 10/22/20	NCT03373877	[95]
1. Siremadlin	1. Inhibits p53-MDM2 interaction	Add on phase 1 parallel design	Incidence DLT within the first 2 cycles; response at the end of 6 cycles—composite of anemia improvement and no spleen volume progression and no symptom worsening	R	NCT04097821 (ADORE trial—platform design)	–
2. Crizanlizumab	2. P-selectin monoclonal antibody					
3. MBG453	3. humanized anti-TIM-3 IgG4 antibody					
9-ING-41	Glycogen Synthase Kinase-3β inhibitor	Add on phase 2	% of patients with response according to the Revised IWG-MRT and ELN Response Criteria for MF (2013)	R	NCT04218071	[96]
Selinexor	nuclear-cytoplasmic transport inhibitor	Second line	Change in spleen volume within 6 months	R	NCT03627403	[97]
Pevonedistat	NEDD8 activating enzyme inhibitor	Add on	Safety and tolerability of the combination as measured by the incidence of AEs and MTD	R	NCT03386214	[98]
Pembrolizumab Nivolumab	PD-1 pathway inhibitors	Second line	Response per ELN-IWG criteria	Completed Terminate	NCT03065400	[99]
					NCT02421354	
AVID200	Selective TGFβ1 ligand trap	Second line Phase 1	MTD and number of patients with response eligibility for Phase 1b	R	NCT03895112	[100]
ONC201	p53 independent promoter of apoptosis	Second-line phase 1	–	–	TBD	[101]
TP3654	second-generation pan-PIM kinase inhibitor	Second-line phase 1	Determine the incidence of DLT and AE	R	NCT04176198	[102]

PIM-Proviral Integration Site for Moloney Murine Leukemia Virus; CDK—Cyclin-Dependent Kinase; IDH—isocitrate dehydrogenase; BH3—B-cell lymphoma 2 (Bcl-2) homology 3; Hsp—heat-shock protein; MDM—murine double minute; TIM—T-cell immunoglobulin and mucin domain; NEDD—Neural precursor cell-Expressed Developmentally Downregulated genes; PD—programmed cell death protein; TGF-transforming growth factor; DLT-dose-limiting toxicity; A-active; NYR—not yet recruiting; R-recruiting; *—in MF pts; SVR35%-35% reduction in spleen volume within 24 weeks; TSS50- ≥ 50% reduction in myelofibrosis-related total symptom score within 24 weeks; AE—adverse events; MTD—maximum tolerated dose

## Conclusion

Advances in diagnostic techniques, i.e., next-generation sequencing, single-cell transcriptome approaches, have carefully refined the molecular signature of MPNs, leading to enhanced insight on clonal dynamics and architecture, thereby informing rationally based treatment approaches. Although HU or IFNa is the front-line agent in the treatment of PV, 25% of patients are intolerant to these agents and experience disease progression while receiving therapy. In light of this, ongoing translational research endeavors have identified mechanistic-based targeted therapeutic agents that may improve the outcomes in PV. Comparably in MF, sustained disease-modifying activity or durable remissions are not seen with the currently approved JAK inhibitors, i.e., ruxolitinib and fedratinib. Therefore, it is crucial to improve upon the existing understanding of the disease and treatment-resistant mechanisms in MF. As such, research efforts are ongoing to develop novel JAK inhibitors or drugs with distinct mechanisms of action that offer a better side effect profile and tolerability in patients with MPNs. Ropeginterferon in low-risk PV, pacritinib in the front-line setting of extreme thrombocytopenia, CPI-0610 combination therapy in JAK inhibitor-naïve patients, imetelstat in the second-line setting to improve survival outcomes, and luspatercept for the treatment of MF patients with anemia are some of the promising agents that look to achieve results in phase 3 trials and gain regulatory approval for the treatment of MPNs.

## Data Availability

Not applicable.

## Abbreviations

JAK: Janus-associated kinase
STAT: Signal transducers and activators of transcription
MPN: Myeloproliferative neoplasms
PV: Polycythemia vera
ET: Essential thrombocythemia
PMF: Primary myelofibrosis
USFDA: The United States Food and Drug Administration
HCT: Hematopoietic cell transplantation
HU: Hydroxyurea
IFNα: Pegylated interferon α
Ropeg: Ropeginterferon alfa-2b
MPD-RC: Myeloproliferative disorders research consortium
CHR: Complete hematological response
HDAC: Histone-deacetylase
TEAE: Treatment-related adverse event
LSD1: Lysine-specific demethylase 1
TI: Transfusion independence
TD: Transfusion dependence
SVR35%: ≥ 35% Spleen volume reduction
IWG-MRT: International Working Group for Myelofibrosis Research and Treatment
BAT: Best available therapy
BMF: Bone marrow fibrosis
TSS50: ≥ 50% Reduction in myelofibrosis-related total symptom score
A: Arm
BET: Bromodomain and Extraterminal domain Protein
PI3K: Phosphatidylinositol 3-Kinase
DLT: Dose-limiting toxicity
ACV: Activin receptor
SMAC: Second mitochondrial activator of caspases
AURKA: Aurora kinase A
TERT: RNA template with reverse transcriptase
